# Finding Mutations That Disrupt Cortical Development

**DOI:** 10.1371/journal.pbio.0020254

**Published:** 2004-08-17

As the presumed “seat of consciousness,” the cerebral cortex mediates the higher-level cognitive processing—such as abstract thought—that humans like to think distinguishes them from other animals. The cerebral cortex is, in fact, significantly larger in the human brain and has far more “columns” than it does in other mammals, particularly compared to the rat, a traditional model for brain study. Neurons in these cortical columns have similar response properties and form fundamental units of brain processing. It is the larger brain surface area, accommodating a greater number of cortical columns, that gives humans the computational edge.

By studying the genetic and molecular agents of cortex development, scientists hope to understand the nature and extent of cortical cognitive function and identify effective therapies to repair brain injury and disease. Analyses of mutant mice have provided insights into the mechanisms controlling cerebral cortex development, but many details of cortical development remain to be revealed. Identifying individual molecules and genes involved in discrete brain processes is particularly difficult given the complexity of brain structure and function. Geneticists have traditionally linked genes to specific biological pathways by first screening large numbers of individuals of a species for unusual physical traits (phenotypes) and then determining the genetic makeup of these mutants to home in on the faulty gene. This approach, called forward genetics, typically requires large numbers of individuals to find unusual phenotypes and so has traditionally focused on fast-breeding organisms like zebrafish and fruitflies. But zebrafish and fruitflies are unlikely to reveal the secrets of higher consciousness.

Now Andrew Peterson and colleagues have updated the forward genetic screen and added a new resource to the neuroscientist's “brain dissection” toolkit. Their approach, which labels specific populations of neurons with protein “reporters,” offers a novel way to find mutations in developing neurons and to identify mutations that interfere with cerebral cortex development. The reporter used here highlights mutations that disrupt interneuron migration into the cortex as well as those that affect cortex growth and morphology. (Interneurons are one of the primary types of cortical neurons.)[Fig pbio-0020254-g001]

**Figure pbio-0020254-g001:**
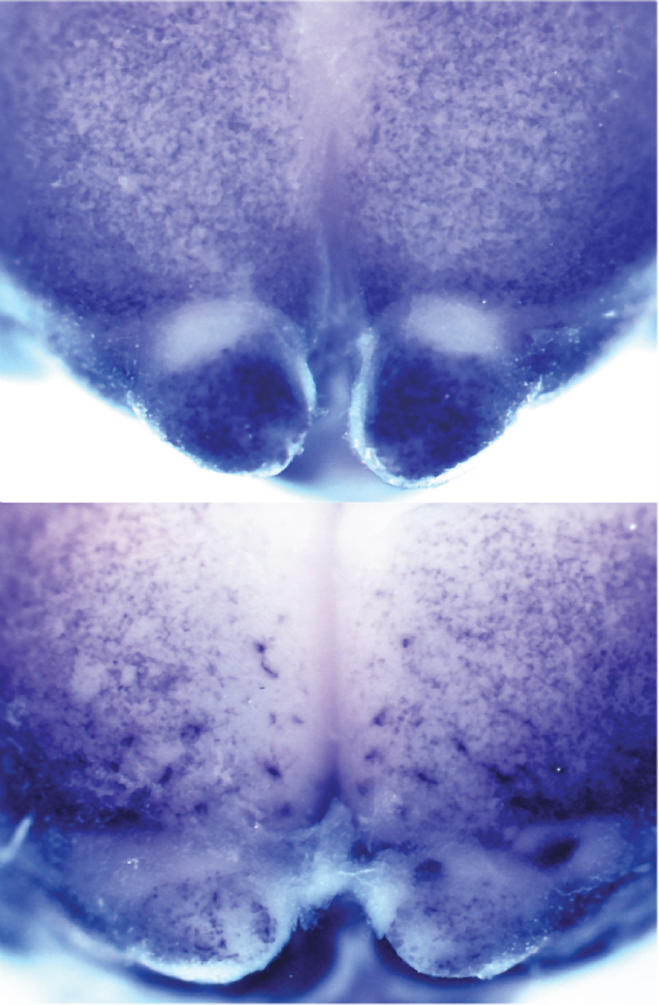
Distribution of GABAergic interneurons in wild-type (top) and mutant (bottom) cortex

Like most forward genetic approaches, the researchers started with a genetically well-characterized breed, then used a chemical mutagen to damage the organism's DNA. After two or three rounds of breeding, the researchers looked for cortical-related defects in the developing embryos. In this case, however, the region of the mouse brain that gives rise to developing interneurons was labeled in the original mice. Thus, when the researchers screened for mutants with defects associated with forebrain development and interneuron migration, they could easily find the cells and genes involved.

The screen identified thirteen mutations affecting cortical development and interneuron migration. (Developing neurons travel along the fibers of other brain cells before reaching their ultimate destination in the brain.) Three mutations are variants of genes known to play a role in cortical development; nine mutations were in genes that had not been linked to cortical development before.

The screen described here takes advantage of a chemical mutagen (called ENU) that induces mutations at a single base pair, or nucleotide, in DNA. These mutations tend to have quite selective effects on protein function—by changing the composition of a single domain—which can provide information on how the protein should normally function and highlight its role in a particular process. The selective nature of ENU, the authors argue, offers new information about how the mutated genes identified here function in cortical development and how these putative roles might be tested.

Altogether, the results suggest that this type of focused screening, so long a resource in fly genetics, can be a powerful tool in mammalian biology as well. That the strategy outlined here could identify novel mutations in a process as complicated as cerebral cortex development suggests that it could do the same for a broad range of biological processes. If the success of other model systems moving in this direction is any indication, this new strategy in the mouse offers researchers a powerful resource for identifying the genetic underpinnings of living systems.

